# Age and estrogen-associated reductions in hypoxic ventilatory response and chemosensitivity in female rats

**DOI:** 10.3389/fphys.2024.1511960

**Published:** 2025-01-28

**Authors:** Jessica M. L. Grittner, Rebecca Barok, Edgar Juarez Lopez, Misha Shah, Brendan J. Dougherty

**Affiliations:** ^1^ Rehabilitation Science Graduate Program, Department of Family Medicine and Community Health, University of Minnesota Medical School, Minneapolis, MN, United States; ^2^ Division of Physical Therapy and Rehabilitation Science, Department of Family Medicine and Community Health, University of Minnesota Medical School, Minneapolis, MN, United States; ^3^ College of Biological Sciences, University of Minnesota, Minneapolis, MN, United States

**Keywords:** hypoxic ventilatory response (HVR), chemosensitivity, estrogen, aging, stepwise hypoxia, respiratory physiology

## Abstract

Respiratory function is modulated by circulating steroid hormones. In female rats, steroid hormones fluctuate across the normal estrous cycle and decline with age, similar to human menopause. To determine the influence of steroid hormones, and mimic aspects of age-related reductions in hormones, the ovariectomy model is often employed. Ovariectomy (OVX) induces an immediate and persistent decrease in steroid hormones. The current study aimed to interrogate whether the OVX model of hormone reduction impacted specific aspects of respiratory function [chemosensitivity and the hypoxic ventilatory response (HVR)] in a manner consistent with natural age-related declines in hormones. Using barometric plethysmography, three experimental groups of female rats were assessed for HVR, chemosensitivity, and respiratory neural drive during progressive hypoxic challenges (FIO_2_: 0.15, 0.12, and 0.09): young (3–5 mos. old; in proestrus; n = 10), young OVX (3–5 mos. old; n = 10), and aged (>20 mos. old; n = 10). Our findings indicted that sex hormone loss did not appear to impact chemosensitivity or neural drive. Natural aging, but not OVX, resulted in decreased HVR as well as reduced magnitude in ventilatory output during stepwise hypoxia. Differences in metabolism were important to the interpretation of these results. Collectively, these data support the concept that aging impacts female respiratory function in complex and unique ways that differ from OVX.

## 1 Introduction

Sex hormones influence respiratory function across the lifespan (Schlenker and Goldman, 1985; Zabka et al., 2003; Zabka et al., 2005; García-Río et al., 2007; Wenninger et al., 2009; Pokorski and Antosiewicz, 2010; Lhuissier et al., 2012; Fournier et al., 2015; Puthon et al., 2017) and affect a variety of mechanisms related to the control of breathing (Behan et al., 2003; Behan and Thomas, 2005; Zabka et al., 2005; Wenninger et al., 2009; McIntosh and Dougherty, 2019; Barok et al., 2021). In humans, there are divergent findings as to whether (or how) hormones impact ventilation (Loeppky et al., 2001; Behan et al., 2003; Behan and Kinkead, 2011) and chemosensitivity (White et al., 1983; Loeppky et al., 2001). Since medical interventions, injury, exposure to endocrine disrupting chemicals, and natural aging all cause substantial changes to endogenous sex hormones, continued study of the hormonal impacts on respiratory function are imperative (Behan and Kinkead, 2011). Unfortunately, comparisons across studies are complicated by the wide variety of data collection and analysis methods used to assess the physiology of this system. Hypoxic ventilatory response (HVR) is one method for assessing the dynamic range and health of the respiratory system, but studies vary in the number, duration, and intensity of the hypoxic stimuli used to assess HVR.

Despite these challenges, a common pattern has emerged for how sex hormones influence respiratory function. Routine changes in circulating sex hormones, such as those observed during the normal menstrual cycles (or the rodent equivalent estrous cycle) in females, have limited impact on resting ventilation or HVR (White et al., 1983; MacNutt et al., 2012; Marques et al., 2017). Early human studies showed that women were more sensitive to hypoxia when circulating estrogen was high (i.e., during the luteal phase of the menstrual cycle; White et al., 1983), but follow-up investigations found large coefficients of variance across experimental groups, and it was posited that HVR is intrinsically variable and prone to sampling error (Lhuissier et al., 2012; MacNutt et al., 2012; Marques et al., 2017). In animal studies, evidence also supports that the estrous cycle exerts minimal influence on HVR (Marques et al., 2015; Marques et al., 2017). Conversely, the dramatic drop in circulating hormones following ovariectomy (OVX) in female rats and cats significantly suppressed the respiratory response to hypoxia in some studies (Tatsumi et al., 1991; Fournier et al., 2015; Marques et al., 2015). Changes related to OVX were typified by reductions in minute ventilation (VE; Fournier et al., 2015; Marques et al., 2015) and significant declines in HVR (Tatsumi et al., 1991), which was defined as the rate-of-change in the relationship between VE and end-tidal PO_2_ (PETO_2_).

The OVX animal model is a useful tool for manipulating sex hormones and is associated with physiological changes similar to those seen with aging (Acosta et al., 2009; Leitner et al., 2009; Rocca et al., 2017), since natural declines in circulating sex hormones occur with aging in females (Burger et al., 2007). These reductions are particularly precipitous following cessation of the estrous/menstrual cycle. However, age-related changes in breathing are multifactorial and likely not the result of one singular variable; age-related changes occur across all physiological domains including the nervous system, endocrine system, muscles, and even lung tissue itself (Sharma and Goodwin, 2006). In human and animal studies, resting ventilation and respiratory neural drive are influenced by aging (Schlenker and Goldman, 1985; García-Río et al., 2007; Wenninger et al., 2009; Pokorski and Antosiewicz, 2010; Lhuissier et al., 2012). Aging also appears to influence HVR (Schlenker and Goldman, 1985; García-Río et al., 2007; Wenninger et al., 2009; Lhuissier et al., 2012; Fournier et al., 2015; Puthon et al., 2017), though specific findings are often contradictory and support the supposition that a wide range of “normal” age-related changes in HVR may be expected (Lhuissier et al., 2012; MacNutt et al., 2012). Collectively, aging studies suggest that group differences in HVR may present only during substantial respiratory duress, like during maximal exercise (Lhuissier et al., 2012; Puthon et al., 2017) or with progressively more severe hypoxic challenges (Schlenker and Goldman, 1985; Morgan et al., 2014). Related to this, some studies have demonstrated that measuring HVR within a single hypoxic episode may not capture the full extent of group differences. In studies that used progressive hypoxia and analyzed the rate-of-change, aging was shown to cause a decline in HVR (García-Río et al., 2007; Wenninger et al., 2009; Pokorski and Antosiewicz, 2010; Lhuissier et al., 2012; Puthon et al., 2017) with a direct relationship to menopause in women (Lhuissier et al., 2012). The relationship between sex hormones, aging, and ventilation are clearly complex, but there is also substantial overlap between these findings and those found in studies using OVX.

Here, we used unrestrained barometric plethysmography to compare ventilation in naturally aged female rats (>20 months old; persistent diestrus) to young OVX female rats and a control group of young, intact female rats during the proestrus phase of the estrous cycle (when circulating estrogen levels are high). Our fundamental question was whether sensitivity of the respiratory system was impacted in a similar manner by two unique models of estrogen loss. In line with prior studies, rats were exposed to progressive levels of inspired hypoxia (FIO_2_: 0.15, 0.12, 0.09) following a normoxic acclimation period. Data was analyzed using rate-of-change analyses allowing for evaluation of HVR, chemoreceptor sensitivity, and respiratory drive (an analogue to neural drive; Morgan et al., 2014). We hypothesized that the OVX and aged groups would demonstrate reduced HVR, neural drive, and chemosensitivity as compared to the young, intact rats. Furthermore, owing to the complex, multisystem effects of progressive aging, we hypothesized that the reductions in HVR and chemosensitivity seen in the aged rats would be greater than those seen in the OVX model.

## 2 Materials and methods

### 2.1 Animals

A total of 30 Fisher 344 rats were used across three experimental groups: young (n = 10), OVX (n = 10), and aged (n = 10). Fisher-344 rats are the considered the “standard” model for investigations of aging given their long lifespan and consistency across colonies (Holmes, 2003). Further, F344 rats are bred by the National Institute on Aging (National Institute on Aging, n.d.) specifically for use in aging-related studies. Here, female Fischer-344 rats were procured from two sources. The aged rats were acquired from the NIA Aged Rat Colony, and rats in the remaining groups (young proestrus and OVX) were purchased from Charles River (Kingston, NJ) which is also the source for the NIA F344 colony. Rats were housed in an AAALAC accredited vivarium; each cage contained two rats with *ad libitum* access to food and water. Light cycles were regulated on a 12:12 h light-dark cycles, shifting phases at 6:00 and 18:00 h. Rats in the young and OVX groups were 3–5 months old and randomly assigned to their groups. The aged rats were >20 months old and determined to be acyclic (i.e., in persistent diestrus; details below) to model a post-menopausal state (Sengupta, 2013). All experimental protocols were approved by the Institutional Animal Care and Use Committee at the University of Minnesota (approval no. 2304-40947A).

### 2.2 Ovariectomy

Surgical procedures were detailed previously (Dougherty et al., 2017; Barok et al., 2021; Miller et al., 2022). Two hours prior to the start of surgery, sustained-release buprenorphine (1 mg/kg; Wedgewood Pharmacy LLC, Laramie, WY, United States) was injected subcutaneously for pain management. Rats were sedated using isoflurane (Piramal, Telangana, India) initially in a closed chamber, and then transferred to a nose cone (2–3% isoflurane in 100% O_2_) for the duration of the surgery. An adequate plane of anesthesia was confirmed by the absence of both toe pinch and eye blink reflexes. Rats were placed prone on a heated surgical table, shaved of fur over the surgical area, and skin scrubbed clean (chlorhexidine scrub). Using a dorsal approach, 10–12 mm bilateral incisions were made through the skin and muscle revealing the ovarian fat pads. Ovaries were exteriorized, removed via cautery, and muscle layers were sutured using 4-0 absorbable suture (Covidien, Mansfield, MA). Skin incisions were sealed using 9 mm wound clips (MikRon, Gardena, CA). Rats recovered in their home cages and were monitored for 72 h for surgical complications. Data collection occurred a minimum of 10 days after OVX.

### 2.3 Determination of estrous cycle

Cytology was used to determine estrous cycle for the young and aged groups. Daily vaginal swabs were performed on all rats for identification of estrous cycle stage. Vaginal cells were examined under light microscopy and cell characteristics and cycle stage determined by two trained judges (Marcondes et al., 2002; Goldman et al., 2007; Dougherty et al., 2017). Data for the young group were gathered during the proestrus phase of the rodent estrous cycle, when circulating estrogen levels are at their highest (Levine, 2015). Both OVX and aged rats were staged to verify cessation of the estrous cycle. Cessation of the estrous cycles was classified as >2 cycles (10 days) of persistent diestrus (Barok et al., 2021). Circulating estrogen levels were subsequently confirmed using an ELISA assay (described below).

### 2.4 Plethysmography

Breathing data was assessed in unanesthetized, freely behaving rats using whole body barometric plethysmography (Buxco, Data Sciences International, St Paul, MN, United States). Bias air flowed at a rate of 2.5 L/min through the 4.0 L chambers, and the pressure was continuously sampled at 500 Hz, temperature, humidity, and barometric pressure were sampled at 10 Hz (Data Sciences International, St Paul, MN, United States). Equipment was calibrated daily per the manufactures instructions to account for fluctuations in barometric pressure (McIntosh and Dougherty, 2019; Barok et al., 2021; Miller et al., 2024). All data was collected during the same time of day (0900-1300) to minimize circadian effects (Stephenson et al., 2001; Marciante et al., 2023). The plethysmography protocol consisted of 1 hour of acclimation, followed by baseline data collection in normoxia (20.9% O_2_, balance N_2_), followed by three 15-minute intervals of stepwise hypoxia, totaling 45 min of hypoxia for each rat (Morgan et al., 2014). The hypoxia steps were as follows: 15% O_2_, balance N_2_, 12% O_2_, balance N_2_, 9% O_2_, balance N_2_. A customizable, gas mixer (GSM-3; CWE, Ardmore, PA, United States) was used to automate the protocol and minimize time between steps. From those traces respiratory frequency (ƒR), inspiratory time (Ti), and expiratory time (Te) were directly recorded. Data was analyzed using Ponemah data acquisition software (Data Sciences International, St Paul, MN, United States). Gas volumes (e.g., tidal volume) were calculated according to the Drorbaugh and Fenn equation, which incorporates chamber and body temperature. Tidal volume (VT) and VE were calculated by Ponemah using temperatures of both the rat and chamber, humidity, and normalized weight per 100 g (Mortola et al., 1994). Body temperature was taken via rectal thermometer just prior to placing the rats in the chambers and immediately following the last hypoxia. Baseline body temperature was used for baseline, FIO_2_ 0.15 and 0.12 calculations; final body temperature was used to for FIO_2_ 0.09 analyses. Five minutes of baseline breathing was selected during a period of rest (not moving, eyes closed) for analysis by an independent analyzer blinded to experimental group designation. Two-minute segments of hypoxic breathing were selected for analysis near the end of each step of hypoxia. During this period of hypoxia, rats were typically awake but holding stable postures. Segments were selected to avoid gas mix transitions and large movement artifacts. Additional, smaller artifacts from sniffing, grooming, or movement were excluded by a trained judge (McIntosh and Dougherty, 2019; Barok et al., 2021; Miller et al., 2024).

Expired gas exiting the chamber was sampled to measure carbon dioxide production (VCO_2_) production. For this, a sampling tube was connected to the expired port of the plethysmograph chamber to divert ∼300 mL/min of gas to a respiratory gas analyzer (Gemini, CWE, Ardmore, PA) via subsampler pumps (Sable Systems, North Las Vegas, NV) and an inline multiplexer (Sable Systems, North Las Vegas, NV). Expired gas data were integrated into Ponemah software to enable real-time VCO_2_ measures throughout the plethysmography sessions. Chambers were not equipped for SpO_2_ acquisition. As such, the current study deviated from Morgan et al. (2014) by using FIO_2_ as a proxy for SpO_2_.

### 2.5 Hormone measurement

Serum estradiol levels were confirmed in a subset of animals from each group (young n = 7, OVX n = 6, aged n = 7). Blood samples were collected after completion of plethysmography data collection. Blood was stored for 24 h at −4°C then centrifuged at 3,000 rpm for 10 min. The serum was stored at −20°C until analysis. Serum levels of estradiol were quantified using ELISA kits from Biovendor (Ashville, rat-estradiol, catalogue no. RTC009R, sensitivity 2.5–1,280 pg/mL). If a sample’s concentration was found to be below the level of detection, a value of 2.5 was entered for analysis. Assays were completed according to manufacturer’s instructions and samples were analyzed in duplicate using a microplate reader (absorbance 450 nm). Concentrations were interpolated from standard curves run in each assay using the microplate reader software (Molecular Devices, San Jose, CA 95134).

### 2.6 Statistical analyses

All statistics were completed using Graphpad Prism software (Dotmatics, San Diego, CA). One-way ANOVAs were conducted to examine differences between group ages, weights, circulating estradiol (E2), and body temperature. One-way ANOVAs were also used to compare baseline measurements of frequency (ƒR), VT, VE, VO_2_, and VCO_2_, neural drive (VT/Ti), and minute ventilation controlling for metabolism (VE/VCO_2_). Responses to hypoxia were analyzed using three methods. In the first approach, two-way ANOVAs were performed for between and within group comparisons of ventilation response (ƒR, VT, VE, VO_2_, VCO_2_, VT/Ti, VE/VCO_2_, and changes to body temperature) across different levels of hypoxia. In the second approach VE, VT/Ti/VCO_2_, and VE/VCO_2_ for each rat were graphed against FIO_2_. Individual slopes were calculated, and the grouped slopes were compared using one-way ANOVA. In the final analysis, the percentage change of output between baseline and maximal hypoxia (FIO_2_ 0.09) was calculated for each animal, and groups were compared using one-way ANOVA. Tukey’s multiple comparisons *post hoc* analyses were conducted when significance was found. Results were considered significant if p-values were less than or equal to 0.05.

## 3 Results

Rats in the young and OVX groups were of similar ages and weights at the time of ventilatory testing. As expected, rats in the aged group were older (p < 0.0001) and heavier (p < 0.0001) than rats in the young and OVX groups (Table 1). Circulating estradiol levels were confirmed and were different between the groups (p < 0.0001). The young, proestrus group had high levels of circulating estrogen compared to OVX (p < 0.0001) and aged (p < 0.0001) groups. The OVX and aged groups did not have significantly different levels of circulating estradiol (p = 0.93). Groups did not differ in core body temperature at the outset of plethysmography testing (p = 0.14). A 2-way ANOVA including pre- and post-plethysmography body temperatures showed a significant main effect of time (p > 0.0001), but there were no significant differences between the groups (p = 0.98). All three groups showed a significant decline in body temperature following exposure to stepwise hypoxia (young p > 0.0001, OVX p > 0.0001, aged p = 0.0001). Post-hypoxia temperatures were similar across groups (Table 1; p = 0.23).

**TABLE 1:** Physiological characteristics of the experimental groups.

Group	n	Age (days)	Weight (g)	Serum estradiol levels (pg/mL)	Baseline body temperature (C)	Post-hypoxia body temperature (C)
Young	10	117.5 (9.05)^†^	156.9 (5.15)^†^	37.67 (5.12)^†#^	37.32 (0.2)	35.84 (0.17)*
OVX	10	135.0 (9.19)^†^	175.8 (4.99)^†^	4.7 (1.45)	37.03 (0.08)	36.11 (0.15)*
Aged	10	675.9 (22.61)	249.6 (7.79)	6.35 (1.03)	36.94 (0.08)	36.16 (0.08)*

Values are mean (SEM). ^†^P < 0.05 vs. aged group. ^#^P < 0.05 vs. OVX group *P < 0.05 vs. baseline for same group. The serum estradiol levels were confirmed using a subset of animals (young n = 7, OVX n = 6 aged n = 7).

### 3.1 Baseline ventilation and respiration

**FIGURE 1:**
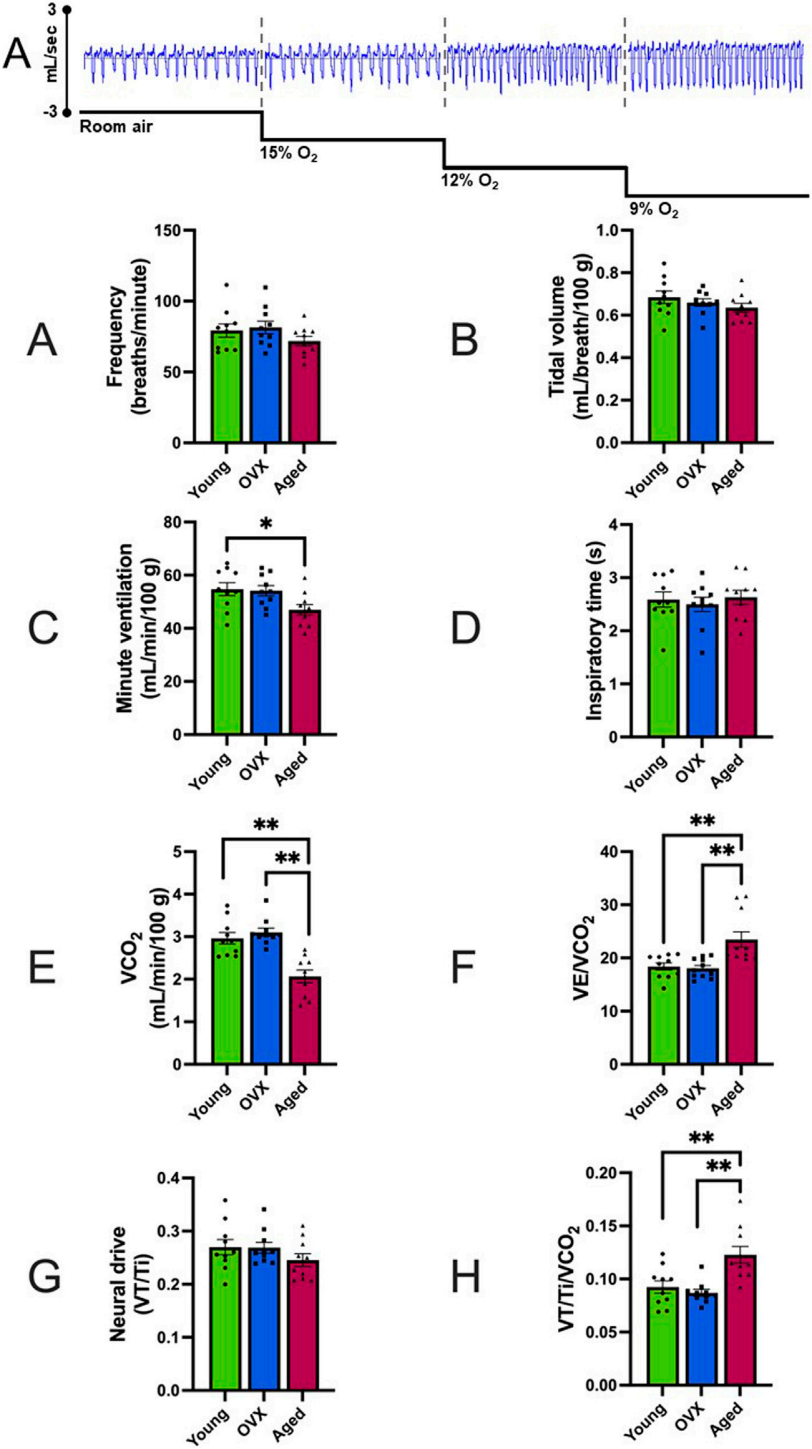
Representative data example and baseline comparisons of ventilation and respiration (mean, SEM). FIO_2_ = 0.21. **(A)** Representative raw data segments from plethysmography pressure sensor data across baseline and stepwise hypoxia collections. In this example, the depth and frequency of the pressure changes show the increased volume and frequency of breathing for this animal. Data presented in Panels **(B–I)** were gathered during the room air portion of the above example. Figure 2 contains data gathered during stepwise hypoxia. **(B)** Breathing frequency in breaths/minute. **(C)** Tidal volume (VT). **(D)** Minute ventilation (VE). **(E)** Inspiratory time in milliseconds. **(F)** VCO_2_ expressed in mL/minute. Significant between-group differences were found in baseline VCO_2_ (p > 0.001), and the aged group had reduced CO_2_ production as compared to the other two groups. **(G)** Minute ventilation controlling for metabolism (VE/VCO_2_) expressed in mL/minute. The aged group demonstrated higher basal ventilation than both other groups. **(H)** Neural drive expressed as tidal volume (VT) divided by inspiratory time (Ti). **(I)** Neural drive controlling for metabolism (VT/Ti/VCO_2_). When metabolism was controlled, the aged group showed an increased neural drive compared to the OVX group. The young group had a large variability and was not significantly different from either group. *p < 0.05, **p < 0.001.

No significant group differences were observed in baseline frequency (Table 2; Figure 1B; p = 0.26), VT (Figure 1C; p = 0.33), or inspiratory time (Figure 1E; p = 0.78). Groups differed significantly in baseline VE (Table 2; Figure 1D; p = 0.03). The young group showed a significantly higher VE compared to the aged group (p = 0.04), and the OVX group had a higher mean VE than the aged group that did not reach significance (p = 0.06). There was a significant group interaction effect for VCO_2_ (p > 0.0001), and post-hoc analyses revealed that the aged group had a significantly decreased VCO_2_ during normoxia compared to both the young (p = 0.0001) and OVX groups (Table 2; Figure 1F; p > 0.0001). We reanalyzed minute ventilation controlling for CO_2_ production (VE/VCO_2_) and found a significant group effect in basal ventilation (p = 0.005); aged rats had significantly higher baseline VE/VCO_2_ compared to the young (p = 0.0024) and OVX groups (Table 2; Figure 1G; 0 = 0.0012). There were no group differences in baseline VT/Ti, which is used as a proxy for respiratory neural drive (Table 2; Figure 1H; p = 0.31). However, when analyzed relative to metabolic rate, a significant group interaction effect emerged. (VT/Ti/VCO_2_; Table 2; Figure 1I; p = 0.0004). Rats in the aged group showed a significantly higher VT/Ti/VCO_2_ compared with young (p = 0.003) and OVX (p = 0.0006) groups.

**TABLE 2:** Ventilatory, metabolic, and temperature data for baseline (0.21 FIO_2_) and stepwise hypoxia conditions.

Variable	Group	FIO_2_			
		0.21	0.15	0.12	0.09
Frequency (breaths/min)	Young	79.27 (4.71)	105.19 (8.01)*	136.86 (5.85)*	151.21 (5.79)*
	OVX	81.41 (4.46)	95.6 (5.74)	151.61 (12.38)*	170.77 (8.81)^†^*
	Aged	71.85 (3.26)	90.37 (5.46)*	123.4 (6.39)*	135.51 (6.82)*
Tidal volume (mL/100 g)	Young	0.68 (0.03)	0.58 (0.03)*	0.51 (0.02)*	0.67 (0.03)
	OVX	0.66 (0.02)	0.6 (0.01)*	0.52 (0.02)*	0.62 (0.03)
	Aged	0.64 (0.02)	0.56 (0.03)*	0.52 (0.02)*	0.62 (0.03)
Minute ventilation (mL/s/100 g)	Young	54.73 (2.44)^†^	62.13 (4.12)	74.48 (4.43)*	107.2 (3.64)^†^*
	OVX	54.15 (1.92)†	59.68 (3.91)	86.06 (6.96)^†^*	112.65 (3.65)^†^*
	Aged	46.97 (1.99)	54.75 (3.53)*	68.96 (2.64)*	87.82 (2.3)*
VCO_2_ (mL/min/100 g)	Young	2.96 (0.14)^†^	2.68 (0.18)	2.53 (0.1)*	2.38 (0.09)*
	OVX	3.1 (0.1)^†^	3.33 (0.16)^†^	2.9 (0.17)	2.74 (0.08)^†^*
	Aged	2.06 (0.15)	2.21 (0.2)	2.07 (0.17)	2.06 (0.1)
Inspiratory time (Ti; ms)	Young	258.75 (0.14)	233.12 (0.18)	170.45 (0.1)*	145.56 (0.06)*
	OVX	249.88 (0.13)	226.86 (0.12)	150.49 (0.11)*	131.36 (0.06)^†^*
	Aged	263.22 (0.13)	209.86 (0.11)	170.76 (0.07)*	158.0 (0.07)*
Neural drive VT/Ti	Young	0.27 (0.01)	0.26 (0.01)	0.31 (0.02)	0.46 (0.02)^†^*
	OVX	0.27 (0.01)	0.27 (0.02)	0.37 (0.02)^†^*	0.48 (0.01)^†^*
	Aged	0.25 (0.01)	0.27 (0.01)*	0.31 (0.01)*	0.39 (0.01)*
VE/VCO_2_	Young	18.59 (0.64)†	24.08 (2.35)	29.55 (1.69)*	45.85 (2.82)*
	OVX	17.48 (0.54)^†^	17.97 (0.89)^†^	30.65 (3.3)*	41.45 (1.77)*
	Aged	23.58 (1.45)	25.63 (1.52)	35.1 (3.0)*	43.59 (2.84)*

Values are means (SEM). FIO_2_, inspired oxygen fraction; VT, tidal volume; VE, minute ventilation; VCO_2_, carbon dioxide production. ^†^P < 0.05 vs. aged group. *P < 0.05 vs. baseline for same group.

### 3.2 Response to stepwise hypoxia

**FIGURE 2:**
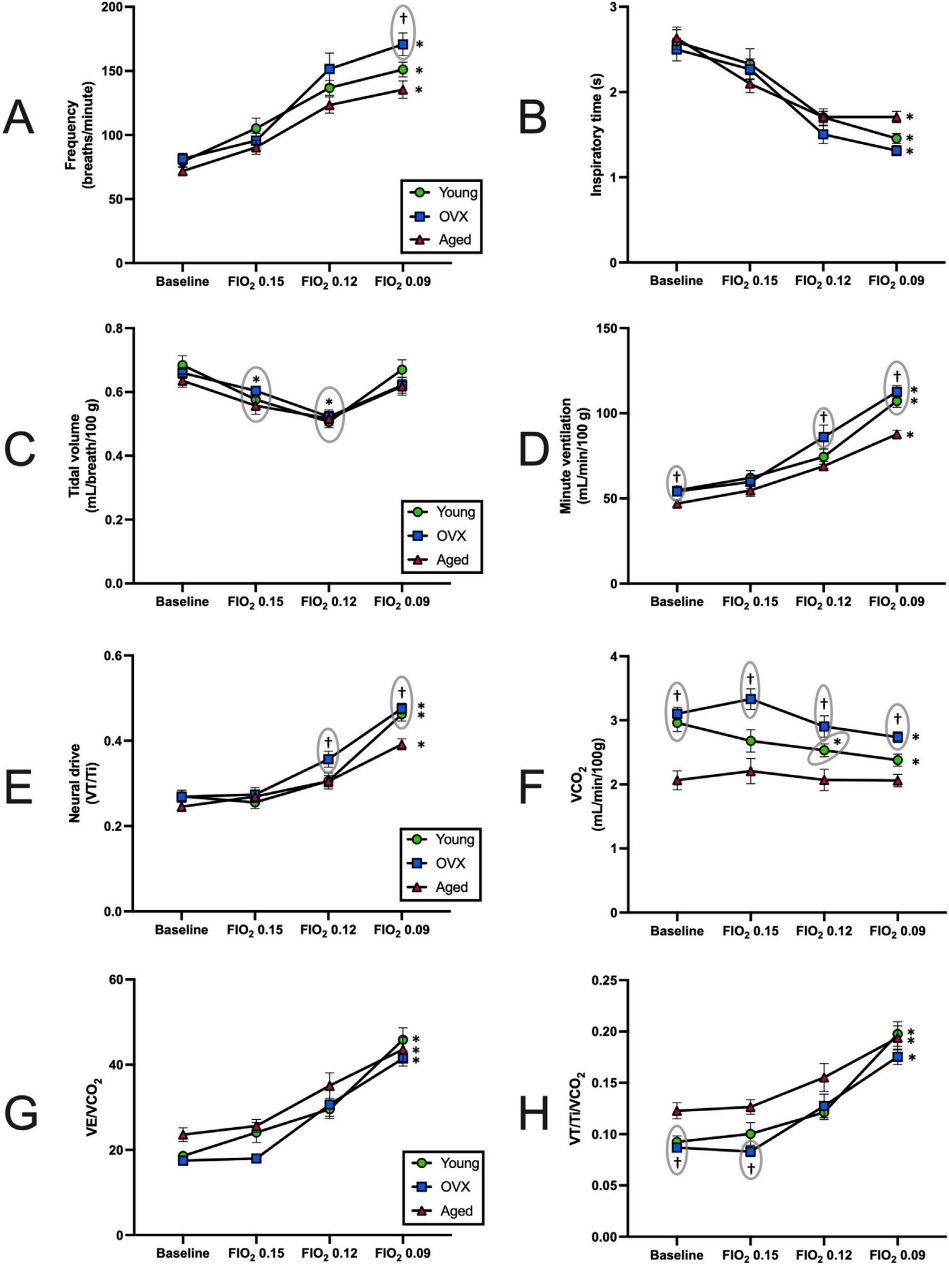
Responses to stepwise hypoxia (mean, SEM). **(A)** Breathing frequency across baseline (BL) and stepwise hypoxia. All groups showed a significant increase in breathing frequency (p < 0.0001). At FIO_2_ 0.09 the OVX group has a significantly higher frequency than the aged group. **(B)** Inspiratory time (Ti) at BL and during exposure to stepwise hypoxia. All three groups significantly decreased in response to hypoxia Ti (p < 0.0001). No between group differences were found. **(C)** Tidal volume (VT), across BL and stepwise hypoxia. All groups had significant declines in VT at FIO_2_ 0.15 and 0.12 before rebounding during maximal hypoxia. There were no significant differences between groups. **(D)** Minute ventilation (VE) across BL and stepwise hypoxia. All groups increased VE in response to hypoxia (p < 0.0001). Young and OVX groups demonstrated larger VE relative to the aged group during baseline and maximal hypoxia. The OVX group also had a significantly higher VE than aged at FIO_2_ 0.12. **(E)** Carbon dioxide production (VCO_2_) during exposure to stepwise hypoxia. The young and OVX groups decreased VCO_2_ output in response to stepwise hypoxia, but the aged group did not change significantly. The aged group had significantly depressed VCO_2_ as compared to the young at baseline, and aged was different from the OVX group in all conditions. **(F)** Minute ventilation controlling for metabolism (VE/VCO_2_), during exposure to stepwise hypoxia. No between group differences were found. **(G)** Neural drive (VT/Ti) at BL and showing significant increases in all groups in response to stepwise hypoxia. The aged group had lower output than the OVX group at FIO_2_ 0.12 and both groups at 0.09. **(H)** Neural drive, controlling for metabolism (VT/Ti/VCO_2_), at BL and during exposure to stepwise hypoxia. All groups significantly increased VT/Ti/VCO_2_ in response to hypoxia. The aged group had a larger output than both groups at baseline and the OVX group at FIO_2_ 0.15. *p < 0.05 as compared to baseline. ^†^p < 0.05 as compared to aged group at same FIO_2_. For clarity, significance markers that appear above data points have a gray circle to indicate which group is included with that marker.

To determine how loss of circulating steroid hormones impacted the HVR and chemosensitivity in female rats, we exposed all three experimental groups to progressive hypoxic challenges (FIO_2_ 0.15, 0.12, and 0.09). Stepwise hypoxia increased ventilation in all experimental groups (Table 2; Figure 2). Breathing frequency showed a significant effect for gas condition (Figure 2A; p < 0.0001) and group (p = 0.014). All groups showed significantly increased rate from baseline to the maximal hypoxic challenge (FIO_2_ 0.09; Figure 2A; young p < 0.0001; OVX p = 0.0001 aged p < 0.0001), and the OVX group had a significantly higher frequency than the aged group only at FIO_2_ 0.09 (p < 0.01). Inspiratory time (Ti) decreased as O_2_ concentration decreased for all groups (Figure 2B; p < 0.0001). There were no between group differences in Ti (p = 0.399).

Significant main effects for gas condition were also observed in measures of tidal volume (Figure 2C; p < 0.0001) with no between group differences (p = 0.58). Post-hoc analyses showed a similar pattern of change across the groups. All three groups demonstrated a significant decline in VT between baseline and FIO_2_ 0.15 and 0.12, but at FIO_2_ 0.09 VT returned to baseline values. Assessment of minute ventilation (VE) showed a significant main effect in response to progressive hypoxia (Figure 2D; p = 0.02). Gas condition caused significant increases in VE for all groups as FIO_2_ declined (p < 0.0001), and a significant group effect was revealed (p = 0.002). Specifically, the aged group displayed smaller VE than the young group and OVX group at baseline (young p = 0.03, OVX p = 0.02) and at FIO_2_ 0.09 (young < 0.001, OVX < 0.0001). At FIO_2_ 0.12, the aged group had a smaller VE than the OVX group (Figure 2D; p = 0.04).

Neural drive (VT/Ti) significantly increased with exposure to hypoxia for all groups (Figure 2E; p < 0.0001). The effect of group was also significant for VT/Ti (p = 0.05). The aged group showed a subdued response compared to the OVX group at FIO_2_ 0.12 (p = 0.04) and compared to both the young and OVX groups during the most severe hypoxia (young p = 0.003, OVX p = 0.0002).

Previous research has strongly suggested that metabolic inhibition occurs during exposure to hypoxia (Gautier and Bonora, 1992; Mortola et al., 1994; Mortola and Maskrey, 2011; Morgan et al., 2014). Our VCO_2_ showed a significant group × condition interaction effect (Figure 2F; p = 0.01), a significant effect of gas condition (p < 0.0001), and a significant group effect (p < 0.0001). The young and OVX groups’ VCO_2_ declined over the course of hypoxia, but the aged group did not change VCO_2_ in response to hypoxia. For the young group, the decline in VCO_2_ reached significance by FIO_2_ 0.12 (p = 0.01), and the OVX group reached significant decline by FIO_2_ 0.09 (p = 0.01). The aged group started with a lower VCO_2_ at baseline as compared to the young (p = 0.0008) and OVX (p < 0.0001) groups. Hypoxia-induced declines in VCO_2_ observed in the young group brought VCO_2_ within range of the aged group at each level of hypoxia (FIO_2_ 0.15 p = 0.19, FIO_2_ 0.12 p = 0.08, FIO_2_ 0.09 p = 0.07). However, VCO_2_ in OVX rats remained higher than the aged group at all data points (FIO_2_ 0.15 p = 0.001, FIO_2_ 0.12 p = 0.006, FIO_2_ 0.09 p = 0.0001).

We next calculated both neural drive and minute ventilation relative to VCO_2_ (VE/VCO_2_ Figure 2G and VT/Ti/VCO_2_ Figure 2H). Our analysis of VE/VCO_2_ revealed that all experimental groups significantly increased VE/VCO_2_ in response to stepwise hypoxia (Figure 2G; p < 0.0001). Although there were baseline differences (noted above, Figure 1C), there were no between group differences over the course of stepwise hypoxia (p = 0.11). All three groups had a significant increase from baseline by FIO_2_ 0.12 and similar patterns of change (Figure 2G).

When metabolism was considered in the context of neural drive, we found a significant main effect (p = 0.04), a significant effect of gas condition (p < 0.0001), and a significant group effect (Figure 2H; p = 0.01). Exposure to stepwise hypoxia increased neural drive for all groups. The increase in VT/Ti/VCO_2_ reached significance by FIO_2_ 0.12 for all groups (Figure 2H; young p = 0.04; OVX p = 0.02; aged p = 0.008). Post-hoc analyses of the group effect showed that the aged group had increased VT/Ti/VCO_2_ relative to the other groups at baseline (young p = 0.02, OVX p = 0.003), and compared to the OVX group at the beginning of stepwise hypoxia (p = 0.0003), but all three groups had similar output as the hypoxia became more severe.

### 3.3 Rate of change & magnitude analyses

**FIGURE 3:**
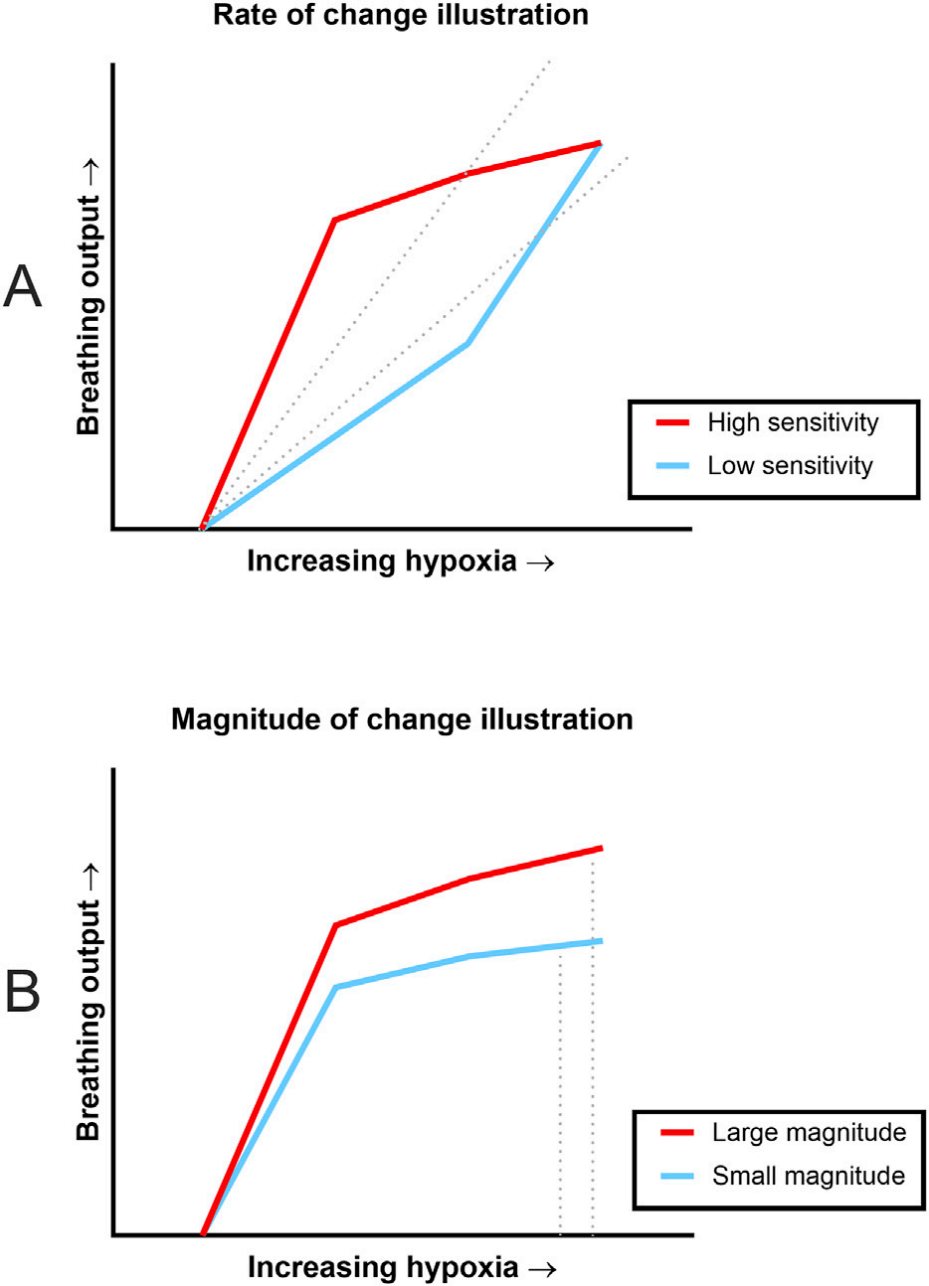
Illustrations of the difference between rate of change and magnitude of change. These illustrations are based on slope and magnitude patterns seen during data analysis. **(A)** Illustration shows differing rates of change in response to stepwise hypoxia but similar magnitudes of response. The red (upper) line signifies data where there was a quick response to increasing hypoxia (high sensitivity) whereas the blue (lower) data set was slower to respond (low sensitivity). Rate of change is condensed into a single number, slope, which is signified by the dotted lines. However, both data sets begin and end at the same points, indicating similar magnitude of change despite very different slopes. **(B)** Illustration shows how small (non-significant) differences in rate of change can result in differing magnitudes of change. Both lines signify data where there were similarly fast responses to changes in oxygen concentration, but the red (top) line shows a larger amount of change from baseline (large magnitude of change) compared to the blue (bottom) line (small magnitude of change). Again, magnitude of change would be condensed into single values based on the dotted lines.

Our fundamental question for the current study was whether sensitivity and dynamic range of the respiratory system was impacted by estrogen loss in two unique experimental models. In other words, we wanted to know whether the rate of change was faster or slower between groups (i.e., sensitivity), and we wanted to know whether the magnitude of change was larger or smaller between groups (i.e., dynamic range). Figure 3 has illustrations based on patterns seen in the rate of change and magnitude of change data. The illustration in Figure 3A shows how two animals could have different rates of change but similar magnitudes of response to hypoxia. The top, red line represents an animal that had immediately increased breathing output in response to increasing hypoxia, whereas the lower, blue line represents an animal that was slower to respond to increasing hypoxia. In other words, the red line shows higher sensitivity to the stimuli than the blue line. This sensitivity is captured by computing the slope of the data (represented as dotted gray lines). For our analyses, the slope (or rate of change) was calculated for each animal and then group analyses were completed. Another aspect of Figure 3A is that both lines begin and end at the same points, showing that even with dissimilar sensitivities to the stimuli, there can still be similar magnitudes of change from beginning to end. Figure 3B illustrates two hypothetical animals that had very similar sensitivity to the stimuli (the slopes of the lines are similar), but the top red line represents an animal that produced a larger amount of change from start to finish (i.e., larger magnitude of change) as compared to the lower blue line. In our analyses, the percent change from baseline to maximal hypoxia was calculated and then group analyses were carried out.

**FIGURE 4:**
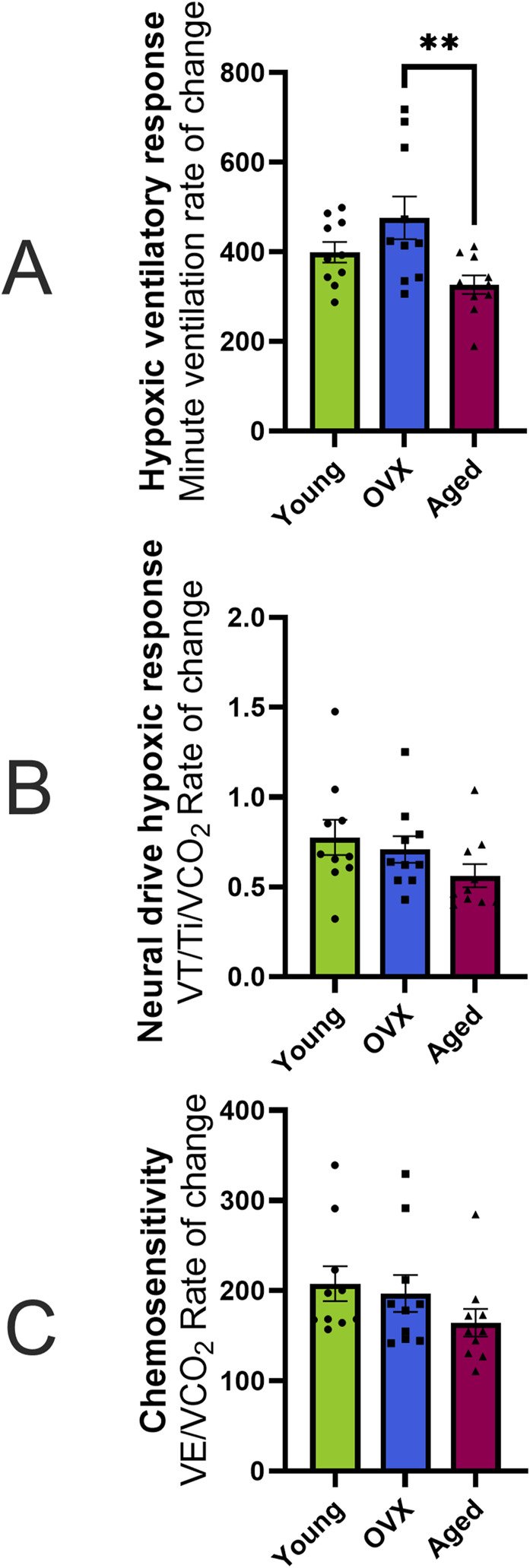
Hypoxic ventilatory response (HVR), neural drive, and chemosensitivity expressed as the rate of change (slope) in response to hypoxia (mean, SEM). **(A)** HVR calculated as the absolute rate of change (i.e., slope) in minute ventilation (VE; mL/min/100 g) across stepwise hypoxias. None of the differences in slope were significantly different. **(B)** Neural drive response to hypoxia calculated as the absolute change in neural drive while controlling for metabolism (VT/Ti/VCO_2_) over the course of stepwise hypoxias. All three groups showed a similar speed in the neural drive response to stepwise hypoxia. **(C)** Chemosensitivity as measured by the absolute change in VE/VCO_2_ across stepwise hypoxias. There were no significant differences in sensitivity between groups. *p < 0.05.

Utilizing the “Gold-Standard” analyses described in Morgan et al. (2014) to assess HVR and chemosensitivity, we defined each animal’s HVR as the slope of VE. Since metabolism is influenced by a variety of factors including age and concentration of sex hormones, and therefore may differ between our groups, we used VCO_2_ as a factor in calculations for neural drive and chemosensitivity. The neural drive response to hypoxia was defined as the slope of VT/Ti/VCO_2_. Chemosensitivity was defined as the slope of VE/VCO_2_ in response to stepwise hypoxia. One-way ANOVAs were used to compare the grouped slopes of respiratory output as the animals were exposed to increasing levels of hypoxia. One-way ANOVA of HVR showed that the rates of change were significantly different between the groups (Figure 4A; p = 0.01). Post-hoc analyses showed that there was a significant difference between the OVX and aged groups (p = 0.01) but not the young and aged groups (p = 0.28). Other results indicated that the aged group showed metabolic differences from the young and OVX groups. Once the individual differences in VCO_2_ measures were included, one-way ANOVAs of neural drive sensitivity (VT/Ti/VCO_2_ slope) and chemosensitivity (VE/VCO_2_ slope) showed no group differences in rates of change (Figure 4B, VT/Ti/VCO_2_ slope p = 0.18; Figure 4C, VE/VCO_2_ slope p = 0.24). Consistent with prior studies (Lhuissier et al., 2012; MacNutt et al., 2012), the variability in these measures was notable, and we opted to show the individual data points for Figure 4 to highlight this range of responses.

**FIGURE 5:**
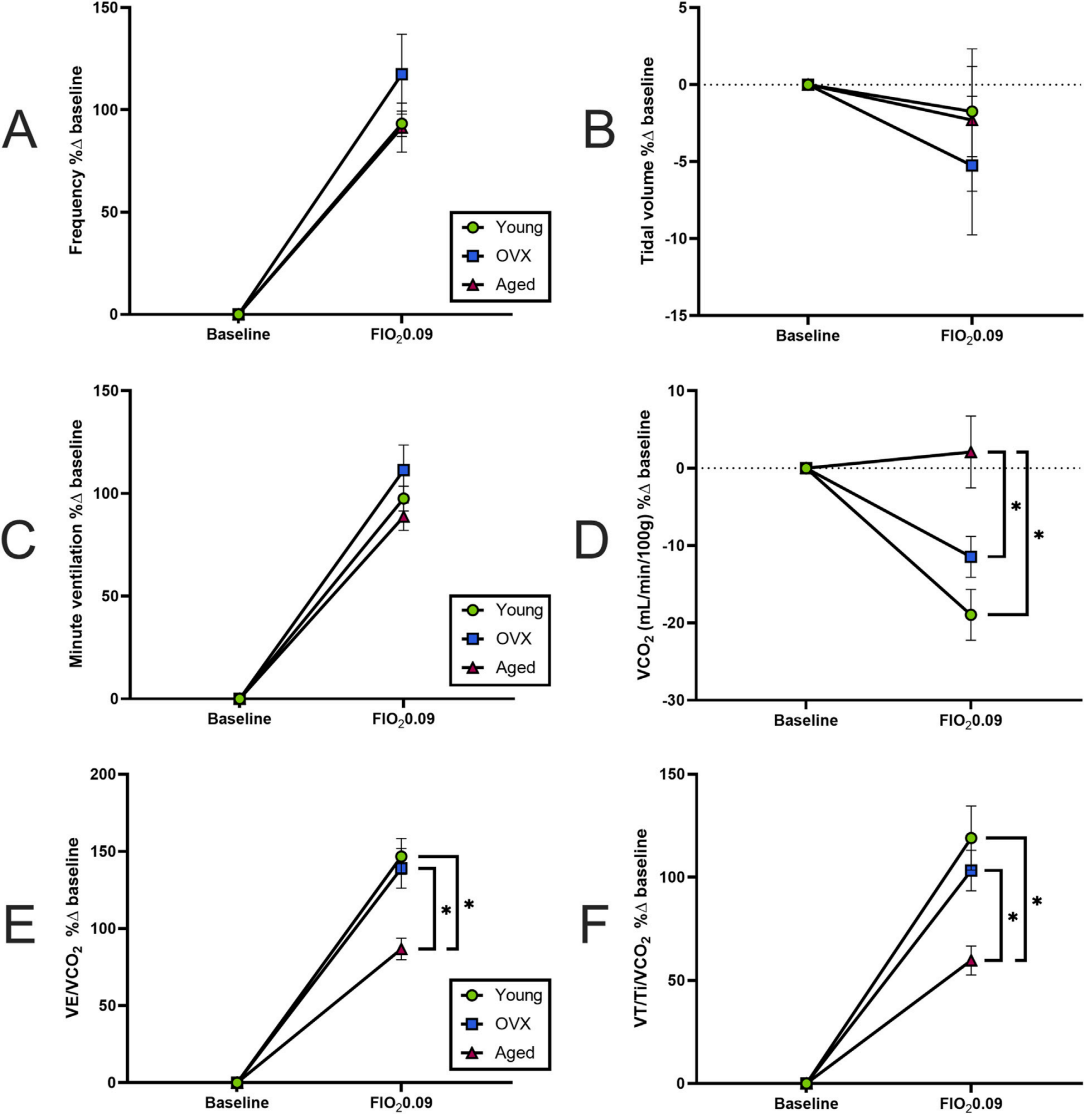
Magnitude of hypoxic responses expressed as percent change from baseline (mean, SEM). **(A)** Frequency (breaths/minute). **(B)** Tidal volume (VT). **(C)** Minute ventilation (VE). All groups increased their frequency, VT, and VE in similar magnitudes. **(D)** Magnitude of VCO_2_ change. As seen in Figure 2F, the aged group had a non-significant, though slightly positive, increase in VCO_2_ whereas the young and OVX groups had (mostly non-significant) decreases in VCO_2_. The result is a large difference in the magnitude of change in response to hypoxia between the aged group and both the young (p = 0.008) and OVX (p = 0.007) groups. **(E)** Magnitude of VE/VCO_2_ change. Although the groups showed similar sensitivity in their rates of change (Figure 4C), the aged group had a smaller magnitude of change compared to both the young (p = 0.05) and OVX (p = 0.018) groups because the aged rats started from a higher of VE/VCO_2_ at baseline (Figures 2G, 3C). **(F)** Magnitude of neural drive, controlling for metabolism (VT/Ti/VCO_2_). Figure 4B showed similar rates of neural drive response to hypoxia. Here the aged group demonstrated a smaller magnitude of VT/Ti/VCO_2_ output in response to stepwise hypoxia. In Figures 2H, 3B, the aged data can be seen starting at a higher output and staying high, resulting in a smaller magnitude of change. *p < 0.05 between groups.

We then compared the magnitude (% change) of responses between baseline and maximal hypoxia (Figure 5). There were no significant group differences in the magnitude of the frequency response to hypoxia (Figure 5A; p = 0.34), VT (Figure 5B; p = 0.81), or VE (Figure 5C; p = 0.2). As suggested by the data in Figure 2F, the magnitude and direction of VCO_2_ changes were significantly different between the aged rats and other groups (Figure 5D; p = 0.001). Accordingly, significant group differences were seen in percent change of VE/VCO_2_ (Figure 5E; p = 0.0009). Although the groups showed similar sensitivity in their rates of change (Figure 4C), the aged group had a smaller magnitude of change because they started from a higher baseline VE/VCO_2_ (Figures 2G, 3C) compared to young (p = 0.002) and OVX (p = 0.005) groups. The magnitude of neural drive change, controlling for metabolism (VT/Ti/VCO_2_; Figure 5F) was significantly different between groups (p = 0.003). Post-hoc analyses showed the aged group had a smaller magnitude of VT/Ti/VCO_2_ change in response to stepwise hypoxia than the young group (p = 0.003) and the OVX group (p = 0.03). Collectively, the aged rats started at a higher output and remained high, resulting in a smaller magnitude of change than the young and OVX groups.

## 4 Discussion

Circulating sex hormones are important for normal respiratory function in females. This supposition is supported by data demonstrating that respiratory-related clinical pathologies (e.g., obstructive sleep apnea) dramatically increase with the natural, age-related transition through menopause; a period typified by the gradual loss of circulating sex hormones (Lin et al., 2007; Bonsignore et al., 2019). Pre-clinical rodent studies exploring the mechanistic impact of reduced circulating hormones commonly involve the removal of the ovaries (OVX) in female rats. This relatively simple surgical procedure provides a fast and reproducible means to diminish circulating hormone levels to assess physiological impact. Though OVX represents a critically important model in this regard, it does not replicate the multisystem impact of aging that includes the gradual loss of circulating hormones in post-menopausal females. This study directly compared the hypoxic sensitivity of both OVX and aged female rats to determine if loss of circulating hormones, regardless of model (natural aging or OVX), influenced hypoxic responses relative to young, ovary-intact female rats. Our chief finding was that the loss of sex hormones does not appear to change chemosensitivity or neural drive responses to stepwise hypoxia. However, aging did reduce HVR as well as the magnitude of ventilatory output in response to stepwise hypoxia, and metabolism was an important consideration in the interpretation of that finding.

During normoxic conditions, all three groups demonstrated similar baseline frequency, VT, inspiratory time, and neural drive (Figures 1A, B, E, H). Following baseline measures, the animals were exposed to multiple levels of hypoxia (FIO_2_ 0.15, 0.12, and 0.09) for analyses that explored rate of change (i.e., sensitivity of the response to a stimuli) versus magnitude of response (i.e., percent change from baseline). All three groups demonstrated a robust and functional response to stepwise hypoxia. Frequency, Ti, VT, and VE all changed significantly (Figures 2A–D). The cumulative interpretation of the VT, VE, and Ti data is that all three groups compensated for reduced ambient oxygen by increasing their VE, and they did so in similar ways. As oxygen decreased, all groups initially compensated with small, fast breathing (decreasing Ti and VT), but with progressively more intense hypoxia, rats increased both the speed and volume of their breaths (decreasing Ti but increasing VT). These findings are consistent with the established literature in females (Lhuissier et al., 2012; MacNutt et al., 2012; Marques et al., 2017). Interestingly, these data differ slightly from the findings of Morgan et al. (2014) who showed concurrent increases in frequency *and* tidal volume in response to stepwise hypoxia. Pattern differences in the hypoxic responses could be due to differences in rat sex or strain; adult, male Sprague-Dawley rats were used in the studies by Morgan et al. Strohl et al. (1997) compared male and female Sprague-Dawley, Brown Norway, and Zucker rats and showed that Sprague-Dawley rats had a slower, deeper breathing patterns compared to the other two strains (Strohl et al., 1997). Future studies comparing male and female responses would add additional data for comparison of sex and strain differences (Schlenker and Goldman, 1985; Mortola and Saiki, 1996; Wenninger et al., 2009; Lhuissier et al., 2012).

A notable exception to the consistencies between our groups was VCO_2_. The aged group showed significantly subdued metabolism compared to the young and OVX groups as demonstrated by a significantly lower VCO_2_ at baseline (Figure 1E) and at many points during stepwise hypoxia (Figure 2F). Morgan et al. (2014) concluded that changes in simple VE account for basic ventilatory responses and neural drive can be inferred by tidal volume/inspiratory time (VT/Ti), but both measures better quantified chemosensitivity when the analysis accounted for metabolism. During hypoxia, changes to metabolism are a key part of the respiratory response. A decline in ambient O_2_ triggers an increase in inhalations to bring more O_2_ into the lungs. However, the increased exhalations lower CO_2_ levels in the blood and upsets acid-base homeostasis unless metabolism is also reduced (for a more thorough summary see Mortola and Maskrey, 2011). Since the release of heat is a final byproduct of cellular metabolism, reduced metabolism manifests as a reduction in core body temperature in response to hypoxia (as seen in Table 1). As such, there is a complicated relationship between VE, temperature, and metabolism to maintain acid-base homeostasis. Within the context of this study, the aged group significantly increased VE in response to hypoxia but did not show significantly decreased VCO_2_. Since the aged rats started from a position of reduced VCO_2_, this finding is not altogether surprising. It has been well reported that aging reduces baseline PaCO_2_ (Hardie et al., 2004) as well as metabolism (Janssen and Ross, 2005; Pataky et al., 2021). It is interesting to speculate that hypoxia may have brought our aged group’s blood PaCO_2_ into the normative ranges of the younger rats or tipped them into acidosis. Either way, age contributed to a less dynamic metabolic response to hypoxia.

Aging related decreases in CO_2_ production is consistent with human studies (Stam et al., 1994; Cardús et al., 1997; Sharma and Goodwin, 2006; Bao et al., 2023; López-Otín et al., 2023) and prior studies in rats (McCarter and Palmer, 1992; Roman et al., 2016). When we accounted for this reduced CO_2_ production of the aged group by dividing VE or VT/Ti by VCO_2_, significant differences emerged. In the end, Morgan et al. advanced that the “best index currently available to quantify peripheral chemoreceptor hypoxic sensitivity is the slope of the ventilatory response over several levels of steady-state hypoxia, of VE/VCO_2_ vs. SpO_2_.” At baseline, the aged group showed higher VE/VCO_2_ (Figure 1F) than the young and OVX groups and a higher metabolism-adjusted neural drive (VT/Ti/VCO_2_; Figure 1H) than the OVX group. Since hypoxia exposure inhibits metabolism (Gautier and Bonora, 1992; Mortola et al., 1994; Mortola and Maskrey, 2011; Morgan et al., 2014), we were interested to see if these metabolic differences would impact the aged group’s sensitivity to hypoxia. Accordingly, we exposed all three groups to stepwise hypoxia. In response to hypoxia, we centered our conclusions around two analyses: rate of change (i.e., the sensitivity of the system in response to hypoxia) and magnitude of change. All three groups demonstrated a prompt response to hypoxia (Figures 2, 4). The OVX group showed an elevated HVR (rate of change in VE) as compared to the aged group, but there were no significant differences in neural drive (rate of change in VT/Ti/VCO_2_) or chemosensitivity (rate of change in VE/VCO_2_). Although all groups demonstrated a robust response to stepwise hypoxia, there were differences in the magnitudes of change for VE/VCO_2_ and VT/Ti/VCO_2_ (Figure 5). The aged group had a significantly smaller magnitude of change in VE/VCO_2_ and VT/Ti/VCO_2_. In fact, the young and OVX groups had nearly twice the magnitude of change in VE/VCO_2_ and over twice the magnitude of change in VT/Ti/VCO_2_ as compared to the aged group. These findings suggest that aged female rats may have unique responses to changes in FIO_2_.

As noted above, one important unanswered question is whether young and aged rats experience similar PaO_2_ levels with stepwise hypoxia. In human studies, hypoxia is often administered with SaO_2_ as a cut-off indicator for true hypoxia level. Gathering continuous SaO_2_ may have improved the accuracy of our hypoxia stimuli if differences between groups were present. Among the many reasons why an aged respiratory system may differ from a young system, is the evidence that carotid bodies degenerate with age (Di Giulio et al., 2023). This degeneration could explain why PaO_2_ levels also decline with age (Hardie et al., 2004) and may impact the relationship between ambient oxygen levels and blood gasses even when VE increases. Continuous PaO_2_ monitoring would have added another layer of specificity to our chemosensitivity measures in this study. Unfortunately, we were unable to measure SpO_2_ or PaO_2_ with our plethysmography system.

GDX surgery has a much more immediate impact on circulating sex hormones than the more gradual age-related declines in hormone production seen with natural aging. How the specific temporal dynamics of these hormonal changes influence respiratory function is unknown, especially in the context of the myriad age-related changes that are specific to the respiratory system such as: loss of respiratory muscle mass (Bordoni et al., 2020) and reductions in alveolar surface area and elasticity (Schneider et al., 2021; Wang et al., 2024). Additionally, though we focused these studies around the influence of estrogen, other sex steroid hormones are significantly affected by OVX and aging. Indeed, progesterone and testosterone are also important to different elements of respiratory control (Behan and Wenninger, 2008; Gargaglioni et al., 2019). Prior work has shown that estrogen plays an important role in respiratory neuroplasticity for females (Dougherty et al., 2017; McIntosh and Dougherty, 2019) and males (Zabka et al., 2006), and estrogen receptors are found throughout brainstem respiratory centers (Behan and Thomas, 2005). Our groups were created with these data in mind. However, our study found that sex hormones were not the primary driver of group differences. Rather, the young and OVX groups were mostly similar in respiratory output and hypoxic responses, and age-related metabolic factors were the primary drivers of group differences.

## 5 Conclusion

In this study the loss of sex hormones, whether through aging or ovariectomy, did not appear to dampen the ability to produce a prompt and functional response to stepwise hypoxia. However, the aged group had diminished HVR and magnitude in VE/VCO_2_ and VT/Ti/VCO_2_ responses. Metabolism was an important consideration in interpreting these findings, as the aged group had a lower overall VCO_2_ compared to the young and OVX groups. Furthermore, although it is well established that hypoxia causes metabolic inhibition (Gautier and Bonora, 1992; Mortola et al., 1994; Mortola and Maskrey, 2011; Morgan et al., 2014), our data indicate that aged female rats did not seem to experience this inhibition. These current data suggest that natural aging reduced the magnitude of neural drive and VE/VCO_2_ response in a unique fashion relative to removal of the ovaries. The differences between our groups were largely mediated through variations in metabolism. Integrating VCO_2_ into calculations showed that the aged group’s relatively low VCO_2_ limited the dynamic range of their response to hypoxia and exacerbated group differences. There is much ground to cover in the study of sex as a biologic factor, and the continuing understanding of sex differences is essential to our future understanding of disease processes and therapeutic interventions.

## Data Availability

The raw data supporting the conclusions of this article will be made available by the authors, without undue reservation.
